# Decoupling the effects of nutrition, age, and behavioral caste on honey bee physiology, immunity, and colony health

**DOI:** 10.3389/fphys.2023.1149840

**Published:** 2023-03-13

**Authors:** Miguel Corona, Belen Branchiccela, Mohamed Alburaki, Evan C. Palmer-Young, Shayne Madella, Yanping Chen, Jay D. Evans

**Affiliations:** ^1^ Bee Research Laboratory, United States Department of Agriculture, Beltsville, MD, United States; ^2^ Sección Apicultura, Programa de Producción Familiar, Instituto Nacional de Investigación Agropecuaria (INIA) Colonia, Montevideo, Uruguay

**Keywords:** *Apis mellifera*, vitellogenin, MRJP1, MFE, insulin, DWV, immune genes, dorsal

## Abstract

Nutritional stress, especially a dearth of pollen, has been linked to honey bee colony losses. Colony-level experiments are critical for understanding the mechanisms by which nutritional stress affects individual honey bee physiology and pushes honey bee colonies to collapse. In this study, we investigated the impact of pollen restriction on key markers of honey bee physiology, main elements of the immune system, and predominant honey bee viruses. To achieve this objective, we uncoupled the effects of behavior, age, and nutritional conditions using a new colony establishment technique designed to control size, demography, and genetic background. Our results showed that the expression of storage proteins, including vitellogenin (*vg*) and royal jelly major protein 1 (*mrjp1*), were significantly associated with nursing, pollen ingestion, and older age. On the other hand, genes involved in hormonal regulation including insulin-like peptides (*ilp1* and *ilp2*) and methyl farnesoate epoxidase (*mfe*), exhibited higher expression levels in young foragers from colonies not experiencing pollen restriction. In contrast, pollen restriction induced higher levels of insulin-like peptides in old nurses. On the other hand, we found a strong effect of behavior on the expression of all immune genes, with higher expression levels in foragers. In contrast, the effects of nutrition and age were significant only the expression of the regulatory gene *dorsal*. We also found multiple interactions of the experimental variables on viral titers, including higher Deformed wing virus (DWV) titers associated with foraging and age-related decline. In addition, nutrition significantly affected DWV titers in young nurses, with higher titers induced by pollen ingestion. In contrast, higher levels of Black queen cell virus (BQCV) were associated with pollen restriction. Finally, correlation, PCA, and NMDS analyses proved that behavior had had the strongest effect on gene expression and viral titers, followed by age and nutrition. These analyses also support multiple interactions among genes and virus analyzed, including negative correlations between the expression of genes encoding storage proteins associated with pollen ingestion and nursing (*vg* and *mrjp1*) with the expression of immune genes and DWV titers. Our results provide new insights into the proximal mechanisms by which nutritional stress is associated with changes in honey bee physiology, immunity, and viral titers.

## 1 Introduction

Approximately 35%–40% of the world’s crop production comes from plant species that depend on animal pollination (Klein et al., 2007) which is carried out primarily by honey bees (Kearns and Inouye, 1998; Lorenzo-Felipe et al., 2020). However, populations of honey bees have experienced a severe decline in recent years worldwide (Vanengelsdorp et al., 2009; Neumann and Carreck, 2010; Hristov et al., 2020). Possible causes for colony losses include the effects of pesticides (Henry et al., 2012), nutritional stress (Naug, 2009; Smart et al., 2016a; Dolezal et al., 2016), the parasitic mite *Varroa destructor* (Amdam et al., 2004; Guzmán-Novoa et al., 2010), and synergistic interactions between Varroa and honey bee viruses (Martin et al., 2012). However, none of these factors has been consistently associated with colony losses (Vanengelsdorp et al., 2009), suggesting the combination of several underlying factors in the occurrence of this phenomenon (Oldroyd, 2007; Ratnieks and Carreck, 2010).

Several studies indicate that nutritional stress significantly contributes to colony losses. First, there is a positive relationship between the area of uncultivated forage land surrounding an apiary and annual colony survival (Naug, 2009; Smart et al., 2016a), suggesting that habitat loss plays a significant role in honeybee colony losses. Second, at individual levels, honey bees from colonies kept in areas of high cultivation exhibited decreased levels of physiological biomarkers associated with nutrition compared with bees kept in areas of low cultivation (Smart et al., 2016b; Dolezal et al., 2016), demonstrating that the availability of floral resources affects the nutritional state of honey bees.

Habitat loss associated with increased use of monocultures reduces both the quality (diversity of plant sources) and the quantity of the pollen collected by the bees (Decourtye et al., 2011). Simultaneous flowering in monocultures provides pollen for a limited period, and hives surrounded by monocultures often experience pollen dearth before and after this period. Pollen is the primary source of proteins and lipids for honey bees (Haydak, 1970; Herbert and Graham, 1992), and pollen from different plants differs in amino acid (Keller 2005; Huang 2012) and fatty acid content (Manning, 2007). Thus, the consumption of pollen from diverse plant sources increases the probability of obtaining the set of nutritional components (e.g., essential amino acids and fatty acids) required for balanced nutrition.

Plasticity is an important feature of honey bee behavioral development (Robinson, 1992). During the first two to three weeks of adult life, workers focus on tasks in the hive, such as brood care (“nursing”); while in the last one to two weeks of life, workers tend to forage for food outside the colony (Winston, 1987; Rueppell et al., 2009). This stereotypical behavioral development can be accelerated or delayed according to colony demography and in response to environmental challenges (Robinson, 1992). Critically, the age at which worker bees switch from nursing to foraging is accelerated in response to stressors such as parasites (Janmaat and Winston, 2000; Goblirsch et al., 2013), pesticides (Colin et al., 2019), and malnutrition (Schulz et al., 1998).

Division of labor in honey bees and other social insects revolves around complex interactions among vitellogenin (*vg*), juvenile hormone (JH), and the insulin/insulin-like growth signaling/target of rapamycin (IIS/TOR) pathway (Corona et al., 2007; Nelson et al., 2007; Ament et al., 2008; Nilsen et al., 2011; Corona et al., 2016). Vg is a yolk protein highly expressed in the fat bodies (Corona et al., 2007), secreted into the hemolymph, and then imported by developing oocytes (Raikhel and Dhadialla, 1992). JH is a multifunctional insect hormone synthesized in the corpora allata. *Methyl farnesoate epoxidase* (*mfe*) encodes for an enzyme that catalyzes the conversion of methyl farnesoate to juvenile hormone III acid, the last step in the JH biosynthesis pathway (Helvig et al., 2004). Honey bee genome harbor two genes encoding for insulin-like peptides (*ilp1* and *ilp2*), expressed in several tissues and organs, including the brain and fat bodies expressed in several tissues and organs, including the brain and fat bodies (Corona et al., 2007; Ament et al., 2008; Nilsen et al., 2011). In workers, negative interactions among *vg*, JH, and insulin-like peptides are associated with task performance. Specifically, *vg* levels are higher in nurses compared with foragers (Engels, 1974; Rutz and Luscher, 1974; Bloch et al., 2002; Corona et al., 2007) while JH (Rutz et al., 1976; Robinson, 1992; Hartfelder and Engels, 1998; Bloch et al., 2002) and insulin-like peptides (Corona et al., 2007; Ament et al., 2008; Nilsen et al., 2011) follow an opposite pattern. Mechanistically, JH inhibits *vg* expression (Rutz and Luscher, 1974; Pinto et al., 2000; Corona et al., 2007), but induces higher brain *ilp1* levels (Corona et al., 2007) and precocious foraging (Jaycox et al., 1974; Robinson, 1992). Reducing *vg* expression by RNA interference also results in higher levels of JH (Nilsen et al., 2011) and precocious foraging (Nelson et al., 2007).

In recent years, the proposal that nutrition is an important factor in regulating the division of labor among social insects has received increased support (Schulz et al., 1998; Blanchard et al., 2000; Toth and Robinson, 2005; Toth et al., 2007; Ament et al., 2008; Ament et al., 2010; Daugherty et al., 2011). Pollen consumption is low in young bees, largest in about 9-day-old nurses, and then declines to minimal amounts in foragers (Crailsheim et al., 1992), which rely primarily on honey intake. Cage experiments showed that young workers eating pollen and sugar have higher abdominal *vg* and low brain *ilp1* levels, while same-age bees fed with only sugar had the inverse pattern (Ament et al., 2008); resembling the expression profiles observed in colony-reared nurses and foragers, respectively (Corona et al., 2007). The effect of pollen on behavioral development could result from its protein content. In support of this view, it has been shown that increased levels of hemolymph amino acids, induced by protein injection, resulted in high vg mRNA levels and delayed foraging (Nilsen et al., 2011).

Although insects lack an acquired immunity, they have developed an efficient innate immune system against a broad variety of pathogens (Lavine and Strand, 2002). Innate immunity has been traditionally divided into humoral and cellular responses (Elrod-Erickson et al., 2000). Humoral immune response is mainly associated with antimicrobial peptides such as *abaecin* (Casteels et al., 1990), *defensin1 (def1)* (Casteels-Josson et al., 1994) and *hymenoptaecin* (*hym*) (Casteels et al., 1993). On the other hand, cellular immunity is mediated by haemocytes and their response such as phagocytosis, nodulation and encapsulation (Strand and Pech, 1995). While phagocytosis is mediated by pathogen recognition proteins (Kocks et al., 2005), nodulation and encapsulation are often accompanied by melanization, a process catalyzed by the pro-phenoloxidase (*PPO*) and pro–phenoloxidase activator (PPOact) enzymes (Söderhäll and Cerenius, 1998; Evans et al., 2006; González-Santoyo and Córdoba-Aguilar, 2012).

Different studies have revealed interactions among immunity, nutritionally-regulated division of labor, and levels of pathogens. First, nutrition affects the expression of immune genes (Alaux et al., 2010; Alaux et al., 2011; Corby-Harris et al., 2014) and susceptibility to different pathogens (DeGrandi-Hoffman et al., 2010; Di Pasquale et al., 2013; Basualdo et al., 2014; Tritschler et al., 2017). Second, infections of pathogens and parasites are associated with high JH, low *vg* levels, and precocious foraging (Goblirsch et al., 2013). Despite these advances, little is known about the proximal molecular mechanisms by which colony nutritional conditions lead to changes in physiology associated with behavioral development, susceptibility to pathogens, and, ultimately, colony survival. This knowledge gap is partly due to the complexity of colony-level experiments that complicate the uncoupling of confounding variables. Here we investigated the impact of pollen restriction on key markers of honey bee physiology, including genes encoding storage proteins and members of the IIS-JH pathway that has been associated with the nutritional regulation of behavioral development. We also investigated the effect of nutritional stress on the expression of immune genes and viral titers to gain insights into the interactions between nutrition, immunity, and disease susceptibility. To achieve these objectives, we uncoupled the effects of behavior, age, and nutritional conditions in colonies of similar size, demography, and genetic background. This study connects poor nutrition with changes in honey bee physiology, immunity and viral disease, expanding mechanistic models that explain colony losses in response to nutritional stress.

## 2 Materials and methods

### 2.1 Experimental colonies

**FIGURE 1:**
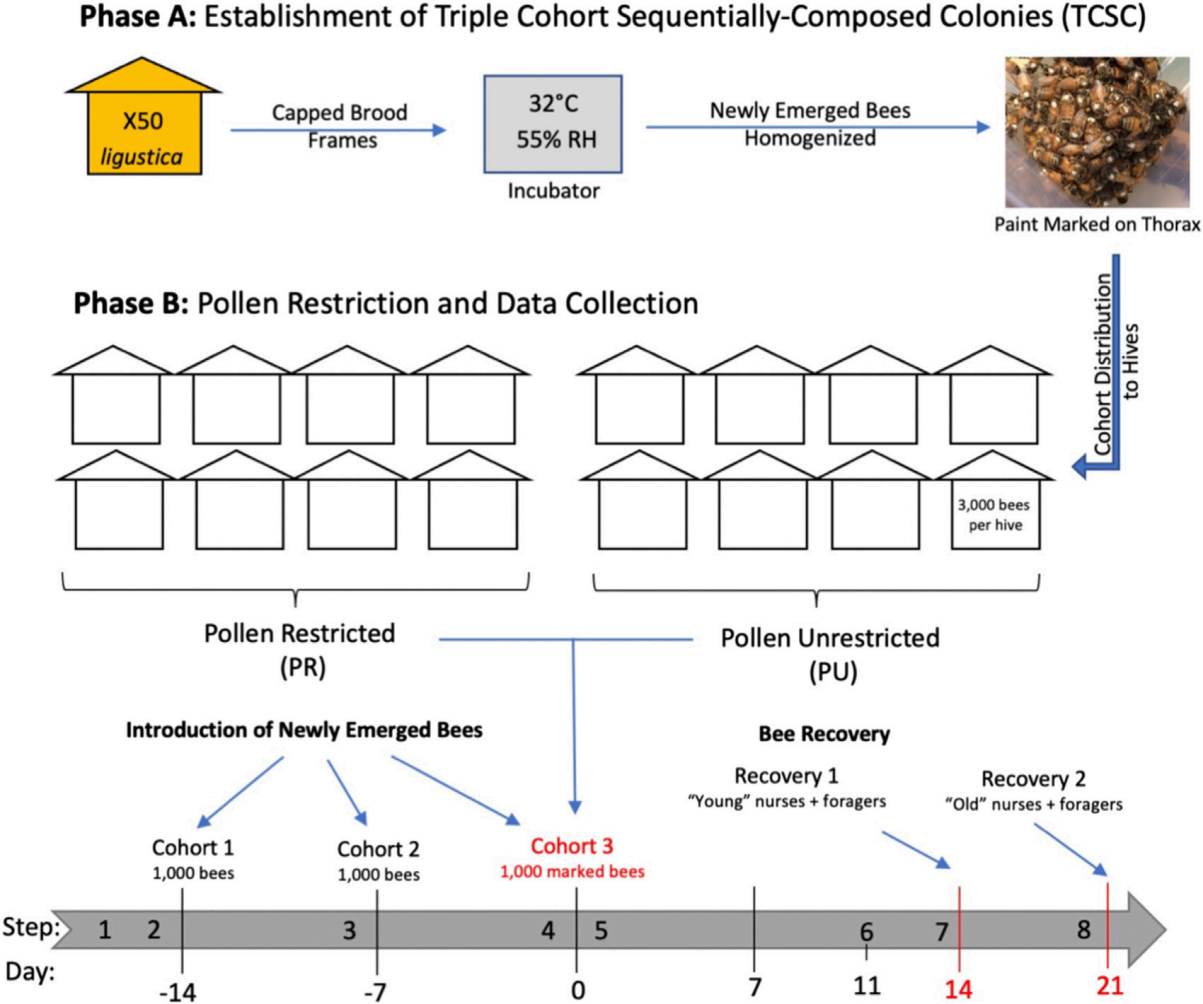
Timeline of experimental design. Numbers below the grey arrow refer to days before and after the end of the colony establishment. Establishment of sixteen triple cohort sequentially-composed colonies (TCSC) using newly emerged bees from 50 Italian colonies. Each TCSC colony consisted of one frame with pollen, one with honey, one empty comb, and one built frame contained in a queen-isolator cage (step 1). One thousand newly emerged bees were introduced into each hive (cohort 1). Sixteen sister queen bees were introduced with cohort 1 and kept confined in queen-insulator cages (step 2). Two subsequent batches of one thousand newly emerging bees were introduced after 1 week (cohort 2) (step 3) and after 2 weeks (cohort 3) (step 4). Two experimental nutritional groups were formed: pollen-restricted (R) and unrestricted (U) colonies (N = 8 per group) (step 5). Pollen restriction was carried out by removing frames containing pollen and activating the pollen traps. Foraging started when bees were 11 days old (step 6). Molecular analyses were conducted on two sets of recovered two-week-old (“young”) (step 7) and three-week-old (“old”) (step 8) nurses and foragers.

Field experiments were performed in the apiaries of the USDA-ARS Bee Research Laboratory in Beltsville, Maryland. The triple cohort composite colony (TCC) is a colony establishment technique designed to vary colony age demography in colonies of similar size and genotypic structure (Giray and Robinson, 1994). A TCC colony is formed by introducing simultaneously three age and behaviorally distinct populations (newly emerged bees, nurses, and foragers) into a colony headed by a queen confined in a push-in wire cage. We modified a previously described protocol (Giray and Robinson, 1994) by sequentially introducing three cohorts of newly emerged bees into a colony headed by a queen confined in a frame enclosed in a queen-excluder cage. This new technique of colony establishment here is called triple cohort sequentially-composite colony (TCSC) (Figure 1).

A set of sixteen colonies, each with a population of three thousand bees, was formed by newly emerged bees from approximately 50 colonies of *Apis mellifera ligustica* and established in four-frame boxes. This experimental design homogenizes the genetic background of the colonies studied: although the genetic variation within colonies is high, variation across the different colonies is expected to be low. For each cohort, newly emerged bees were collected from combs of mature pupae and placed in an incubator at 32 °C and 55% relative humidity. Newly emerged bees were thoroughly mixed, marked on the thorax with enamel paint, and distributed into the colonies. After establishing the colonies, two experimental nutritional groups were formed: pollen restricted (R) and unrestricted (U) colonies (*n* = 8 per group). Subsequent molecular analyses were only performed on recovered bees of the last introduced cohort (cohort 3), (Figure 1).

## 3 Sample collections for molecular analysis

Collections were performed two weeks and three weeks after the last cohort was introduced into the colonies. These collections will be referred to hereafter as young (Y) and old (O). In each of these two age collections, nurses (N) and foragers (F) of the same age were collected from R and U colonies. Therefore, a total of eight age, behavioral and nutritional groups were collected for subsequent analysis. Foragers were collected from the entrance of each colony (double painted) and nurses from the brood area (single painted). Immediately after their collection, bees were flash-frozen in liquid nitrogen for subsequent RNA-extraction and gene expression analyses.

## 4 Gene expression and viral titer analysis

Whole-body bees were homogenized in Trizol (Invitrogen, Waltham, Massachusetts) in a Fast-prep-24 (MP) instrument. Whole bodies were used for analysis because different tissues and organs are expected to participate in the regulation of behavior, immune function and susceptibility to pathogens. RNA extractions were performed with the RNeasy extraction kit and genomic DNA traces were removed using DNAse I (Qiagen, Germantown, MD). RNA was eluted in 100 μL molecular-grade water. For each sample, cDNA was synthesized using 1 μg of total RNA and Thermo Fisher reagents, including M-MLV Reverse Transcriptase (40U), RNase inhibitor (25U), random hexamers (2.5 uM) and dNTPs (0.8 mM) in a final reaction volume of 25 μL. The thermal profile for cDNA synthesis was as follows: 25°C (10 min), 48°C (45 min) and 70°C (5 min). Each cDNA reaction was diluted by adding 100 μL buffer (10 mM Tris HCl pH 8.5). Primers for qPCR analysis were designed using the Oligo 7 program (Rychlik, 2007) (Supplementary Table 1). Transcription levels were quantified by qRT-PCR using a Life Technologies Vii7 system, with SYBR green reagents and a two-step thermal profile for amplification (95°C 15 s, 60°C 1 min). All assays were completed in triplicate in a final volume of 10 μL with no-template and positive controls on each plate. Quantification of mRNA levels was performed by the ΔΔCT method (Livak and Schmittgen, 2001). Relative values were calculated based on the differences (ΔCT) between the CT values of the focal gene/virus and *Rps5* and then transformed to relative values. Data from multiple plates for each focal gene were normalized with a shared reference sample and *Rps5* as an internal control gene (Gregorc et al., 2012). Reference samples were obtained from a pool of concentrated cDNA and placed in each of the 384-well plates used during the qPCR analysis. Data from all the targets was collected and analyzed together using the QuantStudioReal-Time PCR Software v1.3 (https://www.thermofisher.com), which allowed the simultaneous comparison of the levels of different genes and viruses analyzed in this study to be performed. For each comparison, ΔCT values exceeding 2 SD from the mean were considered technical outliers and removed from the analysis. Samples with no amplification of the target were also excluded. Average number of amplified samples after the removal of outliers was as follows: YRN (70), YRF (71), YUN (68), YUF (51); ORN (54), O-R-F (52), OUN (44), OUF (24).

## 5 Statistical analyses

The statistical analyses and figure generations were all carried out in R environment (version 2022.02.3) (Team, 2011). Initial analyses of normality and homoscedasticity of the variables were carried out using the Kolmogorov-Smirnov and Levene tests (Fox et al., 2011; Gross and Ligges, 2015; RStudio Team, 2016). Differences between groups of variables fulfilling the assumptions of parametric statistics were analyzed with ANOVA. Non-parametric variables were analyzed by Mann Whitney U test *a posteriori* (de Mendiburu, 2016). “Behavioral,” “Age,” and “Nutrition” pairwise comparisons were made between treatment combinations. Correlations in the expression level of the different genes and viruses were analyzed by the Spearman Rank Correlation Test (de Mendiburu, 2016; RStudio Team, 2016). Spearman correlations were used to construct a dissimilarity matrix (dissimilarity = 1—correlation coefficient). The dissimilarity matrix was used as input for hierarchical clustering (R function *hclust*) by the complete linkage method. Results of the clustering algorithm were used to create a dendrogram for the visualization of the relationships between different gene targets. Additional correlation analyses were conducted between genes of each treatment category independently using “corrplot” and “ggpubr” libraries with the Spearman method as the data failed the assumption of normality assessed by the Shapiro test. Principal component analysis (PCA) was conducted using the overall average expressions of the studied genes. PCA was carried out using the “factoextra” library to estimate similarity among treatments and expression of each variable on a 3-dimensional scale. Heatmaps were generated using the “pheatmap” library based on the overall average expression of each gene similar to the PCA. Quantifications of two viruses (BQCV and SBV) were excluded from the analysis due to low prevalence. The 205 samples with successful quantification of all 15 remaining molecular targets were used for ordination and statistical testing. Young workers: RN (*n* = 31), RF (*n* = 41); UN (*n* = 24), UF (*n* = 22). Old workers: RN (*n* = 27), RF (*n* = 23); UN (*n* = 27), UF (*n* = 10). Effects of behavior, age, and nutrition on expression were tested by permutational MANOVA (*adonis* function in package *vegan*) (Oksanen et al., 2017).

## 6 Results

## 6.1 Genes associated with physiological activity

### 6.1.1 Genes encoding storage proteins

**FIGURE 2:**
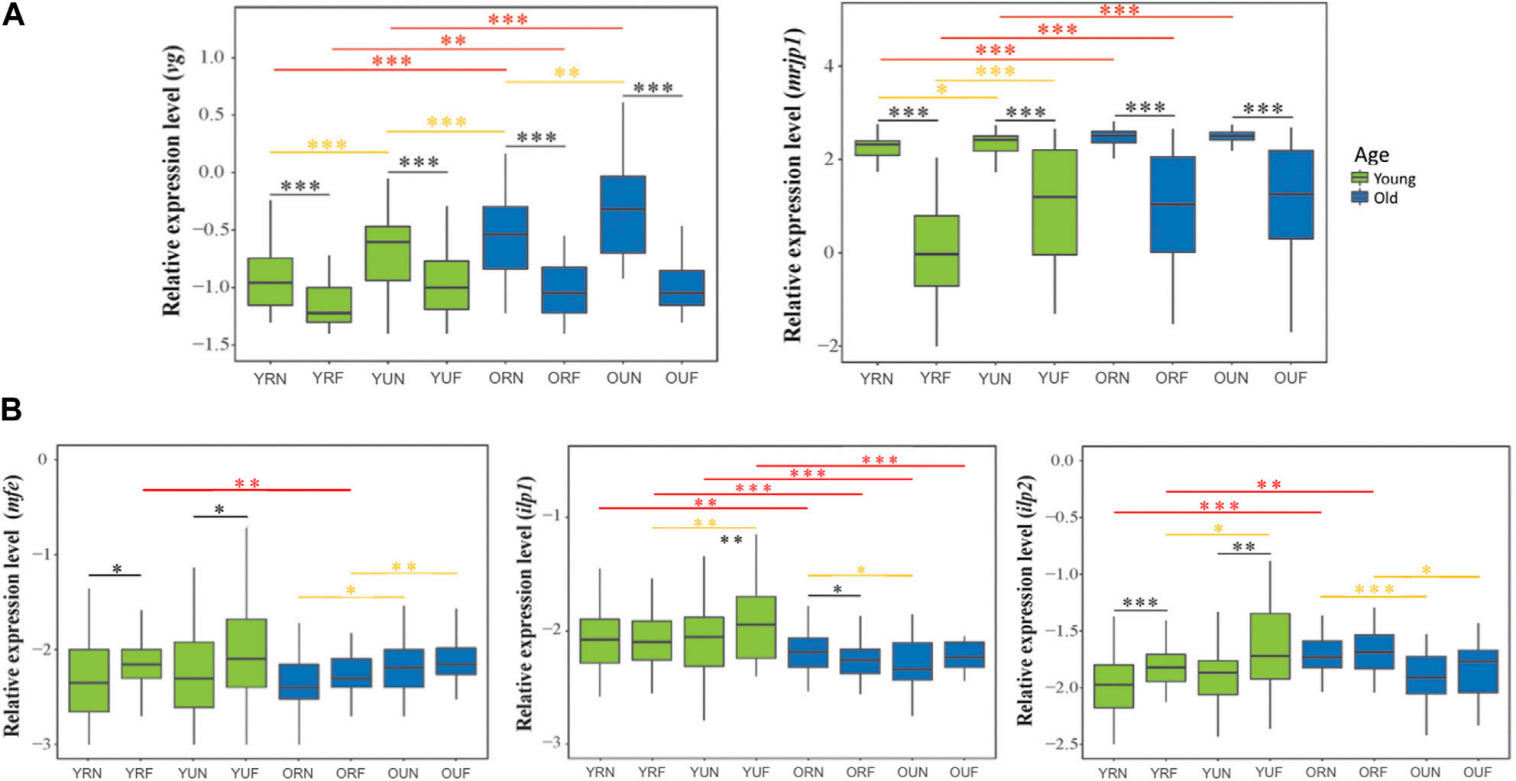
Relative mRNA levels of genes associated with physiological activity. The upper panel **(A)** compares the expression of genes associated encoding storage proteins. The lower panel **(B)** compares the expression of IIS-JH genes involved with hormonal regulation. The *y*-axes indicate log10-transformed relative expression levels. The *x*-axes show the age/nutritional/behavioral groups: young (Y), old (O), nurses (N), foragers (F), pollen-restricted (R) and (U) pollen-restricted. The age of bees referred to as “young” and “old” is 2-week-old and 3-week-old, respectively. Ribosomal protein *Rps5* was used as an internal control. Boxes show the first and third interquartile ranges. A line represents the median. Whiskers include the values of 90% of the samples. Black, yellow and red asterisks illustrate significant differences between behavioral, nutritional, and age groups. *p < 0.05; **p < 0.01. ***p < 0.001.

**FIGURE 3:**
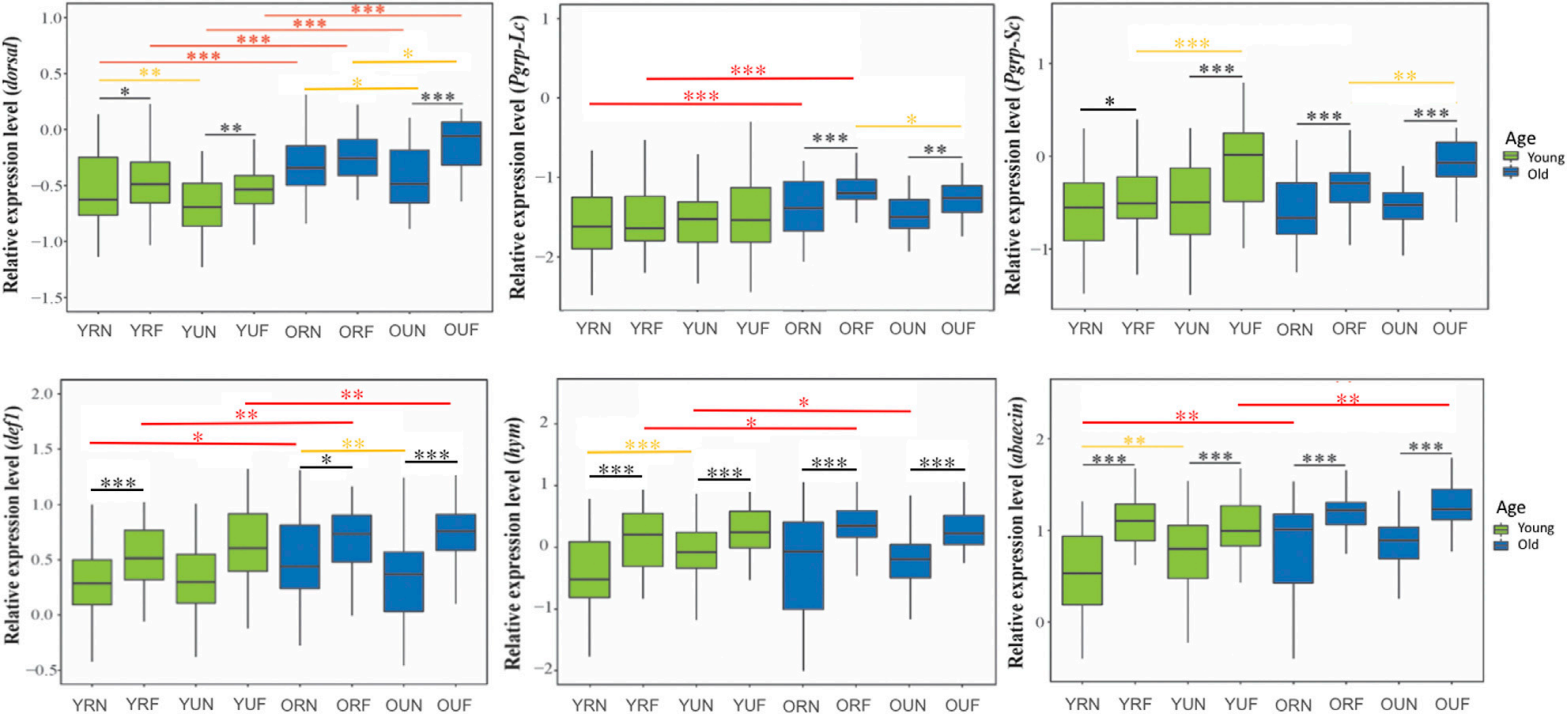
Expression of immune genes involved in the humoral immune response and the regulatory gene *dorsal*. Sample notation is as in Figure 2. Black, yellow and red asterisks illustrate significant differences between behavioral, nutritional, and age groups. *p < 0.05; **p < 0.01. ***p < 0.001.

The expression of the genes encoding for storage proteins (*vg* and *mrjp1*) revealed similar expression patterns: Behavior had a highly significant effect on the levels of *vg* and *mrjp1* (*p* < 0.001) in all the age and nutritional groups; with higher levels in nurses compared with foragers (Figure 3A; Table 1, Supplementary Table 2). Similarly, nutrition affected the overall expression of both *vg* and *mrjp1* (*p* < 0.001), but with some differences associated with age and behavior. For example, *vg* showed higher levels associated with pollen ingestion in young nurses and foragers (*p* < 0.001) and old nurses (*p* = 0.008). In contrast, *mrjp1* showed higher levels associated with pollen ingestion only in young workers (nurses: *p* = 0.01, foragers: *p* < 0.001) (Table 2, Supplementary Table 2). Similarly, age had a significant effect on the overall expression of *vg* and *mrjp1* (*p* < 0.001), with significantly higher levels in older workers in most groups, including nurses and foragers from pollen-restricted colonies (R) and nurses of pollen-unrestricted colonies (U) (Figure 2A; Table 2, Supplementary Table 4). In addition, our whole-body analyses revealed that average *mrjp1* mRNA levels were over 2.5 orders of magnitude higher than *vg* (Figure 2, Supplementary Table 3).

**TABLE 1:** Probability values of the overall effect of experimental variables on gene expression and viral titers. All the targets were analyzed using the Mann-Whitney test. Significant *p*-values are indicated in red case.

	Targets	Behavior	Nutrition	Age	Nut/Beh	Nut/Age	Beh/Age	Nut/Beh/Age
Physiological activity	*Vg*	<0.001	<0.001	<0.001	<0.001	<0.001	<0.001	<0.001
	*mrjp1*	<0.001	<0.001	<0.001	<0.001	<0.001	<0.001	<0.001
	*mfe*	<0.001	0.049	0.07	<0.001	0.015	<0.001	<0.001
	*ilp1*	0.453	0.203	<0.001	0.033	<0.001	<0.001	<0.001
	*ilp2*	<0.001	0.071	0.003	<0.001	<0.001	<0.001	<0.001
Immune genes	*dorsal*	<0.001	<0.001	<0.001	0.061	0.083	0.419	0.043
	*pgrp-lc*	<0.001	0.258	<0.001	<0.001	<0.001	<0.001	<0.001
	*pgrp-sc*	<0.001	0.01	0.963	<0.001	0.013	<0.001	<0.001
	*def1*	<0.001	<0.001	<0.001	<0.001	0.004	<0.001	<0.001
	*hym*	<0.001	0.852	0.179	<0.001	0.005	<0.001	<0.001
	*abaecin*	<0.001	0.973	<0.001	<0.001	<0.001	<0.001	<0.001
	*eater*	<0.001	0.095	0.095	0.597	0.01	0.003	0.016
	*ppo*	<0.001	0.161	0.032	0.604	0.083	0.663	0.010
	*ppo-act*	<0.001	0.454	<0.001	0.130	0.257	0.287	0.028
Viruses	*DWV*	<0.001	0.674	<0.001	<0.001	<0.001	<0.001	<0.001
	*BQCV*	0.027	<0.001	0.454	<0.001	<0.001	0.173	<0.001
	*SBV*	0.111	0.645	0.003	0.01	0.031	0.003	0.002

**TABLE 2:** Probability values of paired comparisons of behavioral, age, and nutritional groups on gene expression and viral titers. All the targets were analyzed using the Mann-Whitney test. Significant p-values are indicated in red case.

		Behavior				Nutrition				Age			
		Young		Old		Young		Old		Restricted		Unrestricted	
		YR	YU	OR	OU	YN	YF	ON	OF	RN	RF	UN	UF
	Targets	YRN/YRF	YUN/YUF	ORN/ORF	OUN/OUF	YRN/YUN	YRF/YUF	ORN/OUN	ORF/OUF	YRN/ORN	YRF/ORF	YUN/OUN	YRF/ORF
Physiological activity	*Vg*	<0.001	<0.001	<0.001	<0.001	<0.001	<0.001	0.008	0.772	<0.001	0.003	<0.001	0.660
	*mrjp1*	<0.001	<0.001	<0.001	<0.001	0.01	<0.001	0.519	0.463	<0.001	<0.001	<0.001	0.584
	*mfe*	0.013	0.018	0.084	0.177	0.793	0.391	0.031	0.006	0.558	0.003	0.551	0.633
	*ilp1*	0.608	0.008	0.091	0.109	0.854	0.001	0.027	0.306	0.005	<0.001	<0.001	<0.001
	*ilp2*	<0.001	0.001	0.455	0.196	0.178	0.01	<0.001	0.04	<0.001	0.001	0.61	0.064
Immune genes	*dorsal*	0.049	0.003	0.157	<0.001	0.009	0.133	0.048	0.021	<0.001	<0.001	<0.001	<0.001
	*pgrp-lc*	0.15	0.536	<0.001	0.002	0.145	0.459	0.088	0.031	<0.001	<0.001	0.392	0.061
	*pgrp-sc*	0.036	<0.001	<0.001	<0.001	0.308	<0.001	0.588	0.002	0.957	0.06	0.403	0.732
	*def1*	<0.001	0.270	0.021	<0.001	0.362	0.092	0.002	0.387	0.005	0.022	0.952	0.004
	*hym*	<0.001	<0.001	<0.001	<0.001	<0.001	0.372	0.539	0.304	0.108	0.029	0.037	0.78
	*abaecin*	<0.001	<0.001	<0.001	<0.001	0.003	0.437	0.64	0.141	0.004	0.105	0.471	0.004
	*eater*	<0.001	<0.001	0.045	<0.001	0.059	0.904	0.165	<0.001	0.413	<0.001	0.394	0.334
	*ppo*	>0.001	0.348	0.008	<0.001	0.77	0.038	0.134	0.009	0.301	0.035	0.003	0.011
	*ppo-act*	<0.001	0.243	0.017	0.049	0.014	0.084	0.186	0.603	0.366	<0.001	<0.001	0.077
Viruses	*DWV*	<0.001	0.778	<0.001	<0.001	<0.001	0.871	0.351	0.144	0.068	0.088	<0.001	0.015
	*BQCV*	0.948	0.092	0.041	0.024	<0.001	0.106	0.052	0.487	0.165	0.628	0.813	0.187
	*SBV*	0.037	0.167	0.434	0.004	0.109	0.035	0.062	0.373	0.707	0.006	0.813	0.161

### 6.1.2 Genes involved with hormonal regulation

Next, we analyzed the effect of pollen restriction on the expression of three key genes of the IIS-JH pathway: *methyl farnesoate epoxidase* (*mfe*), *insulin-like peptide 1* (*ilp1*) and insulin-*like peptide 2* (*ilp2*). We found that behavior significantly affected the overall expression of *mfe* and *ilp2* (*p* < 0.001) (Table 1), with higher levels in foragers (Figure 2B). However, age comparisons revealed that this effect was only significant in young workers (*mfe*: R *p* = 0.013, U, *p* = 0.018; *ilp2*: R *p* < 0.001, U *p* = 0.001). On the other hand, although behavior did not significantly affect the overall expression of *ilp1* (Table 1), this gene was significantly upregulated in young foragers from colonies not experiencing nutritional stress (*p* = 0.008) (Table 2, Supplementary Table 2).

Nutrition had a significant effect on the expression of *mfe* (*p* = 0.049). Although nutritional status did not affect the overall expression of *ilp1* and *ilp2* (Table 1), our analyses revealed significant interactions with age and behavior (Table 2). For example, *ilp1* and *ilp2* showed higher levels in young foragers from colonies not experiencing nutritional stress (*ilp1 p* = 0.001; *ilp2 p* = 0.01). *Mfe* also exhibited a trend toward high levels in young foragers from colonies not experiencing nutritional stress, but significantly higher levels associated with pollen ingestion were found only in old workers (Figure 2B; Table 2). Our results also showed that, in contrast with *mfe*, pollen restriction induced high levels of *ilp1* (*p* = 0.027) and *ilp2* (*p* < 0.001) in old nurses.

On the other hand, age significantly affected the overall expression of *ilp1* (*p* < 0.001) and *ilp2* (*p* < 0.001), but did not show a significant effect on the overall expression of *mfe* (*p* = 0.07). However, *mfe* and *ilp1* showed similar expression patterns, with lower levels in all the groups of older workers (Figure 2B). In contrast, *ilp2* expression increased with age in restricted workers (*p* = 0.001). Additionally, our qPCR analyses revealed that while *mfe* and *ilp1* had an average expression ratio of 1:1, *ilp2* expression is about two-fold higher compared with them (Figure 2B, Supplementary Table 3).

## 6.2 Immune genes

**FIGURE 4:**
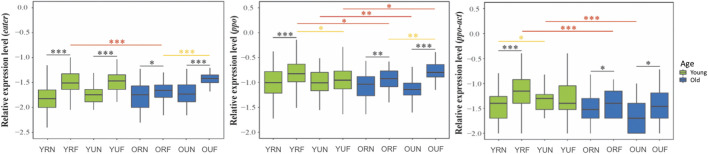
Expression of genes involved with the cellular immune response. Sample notation is as in Figure 2. Black, yellow and red asterisks illustrate significant differences between behavioral, nutritional, and age groups. *p < 0.05; **p < 0.01. ***p < 0.001.

We analyzed the effect of behavior, nutrition, and age on nine representative immune genes: *dorsal*, *def1, abaecin*, *hym*, *pgrp-lc*, *pgrp-sc*, *eater*, *ppo-act*, and *ppo*. Our results show that behavior has a strong effect (*p* < 0.001) on the expression of all tested immune genes, with higher levels in foragers compared with nurses (Figure 3). Gene-specific analyses show that the immune genes more affected by behavior include two genes encoding antimicrobial peptides (*abaecin* and *hym*) (Figures 3, 4; Table 2).

In contrast with behavior, the effect of nutrition was only significant on the expression of the regulatory gene*dorsal* (*p* < 0.001) and two genes involved in the humoral immune response (*def1 p*<0.001, *pgrp-sc p* = 0.01) (Table1). However, aired comparisons among experimental groups showed that nutrition had a statistically significant effect on the expression of immune genes in 13 out of 36 comparisons, with a higher percentage of these comparisons showing significant upregulation associated with pollen ingestion (61.5%) compared with pollen restriction (38.5%). In addition, our analysis also revealed that *dorsal* was the immune gene whose expression was more significantly affected by nutrition (Table 2, Supplementary Table 2).

On the other hand, age significantly affected the expression of most immune genes, except *eater*, *hymenoptaecin, and pgrp-sc* (Table 1, Supplementary Table 2). Gene-specific analyses showed that *dorsal* and the genes involved in the humoral immune response tend to increase with age (Figure 3). On the contrary, the expression of cellular immune genes shows a trend toward downregulation with age (Figure 4).

## 6.3 Virus titers

We analyzed the effects of behavior, nutrition, and age on the titers of three of the more prevalent bee viruses, namely, the Deformed wing virus (DWV), Black queen cell virus (BQCV), and Sacbrood virus (SBV). Our overall analysis of viral titers showed that DWV was the virus most affected by behavior (*p* < 0.001), followed by BQCV (*p* = 0.027) (Table 1). Our results also show that BQCV was the only virus affected by nutrition (*p* < 0.001). On the other hand, we found that age had a significant effect on the titers of DWV (*p* < 0.001) and SBV (*p* = 0.003).

**FIGURE 5:**
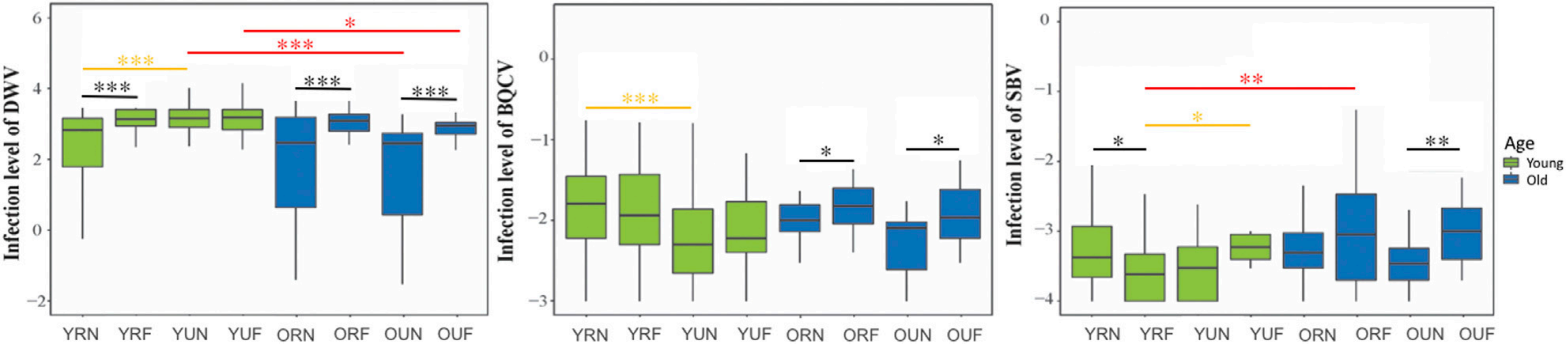
Relative levels of honeybee RNA viruses. The *y*-axes indicate log10-transformed relative levels of DWV, SBV and BQCV relative to *Rps5* (control) gene mRNA level. Sample notation is as in Figure 2. Black, yellow and red asterisks illustrate significant differences between behavioral, nutritional, and age groups. *p < 0.05; **p < 0.01. ***p < 0.001.

Paired comparisons of behavioral, nutritional, and age groups showed that DWV had a strong trend toward higher titers in foragers, with highly significant differences in all nutritional and age groups (*p* < 0.001) except young foragers from U colonies (Table 2). Subsequent nutritional comparisons revealed significantly higher titers of DWV in younger nurses from U colonies (*p* < 0.001). In contrast, we found higher BQCV titers in young nurses from U colonies. Finally, age comparisons revealed a trend to lower titers of DWV in older workers, although significant differences were only observed in workers from U colonies. Also, in contrast with DWV, SBV titers were significantly higher (*p* = 0.006) in older foragers from R colonies (Figure 5; Table 2, Supplementary Table 2).

## 6.4 Correlations within categories of genes and viruses

**FIGURE 6:**
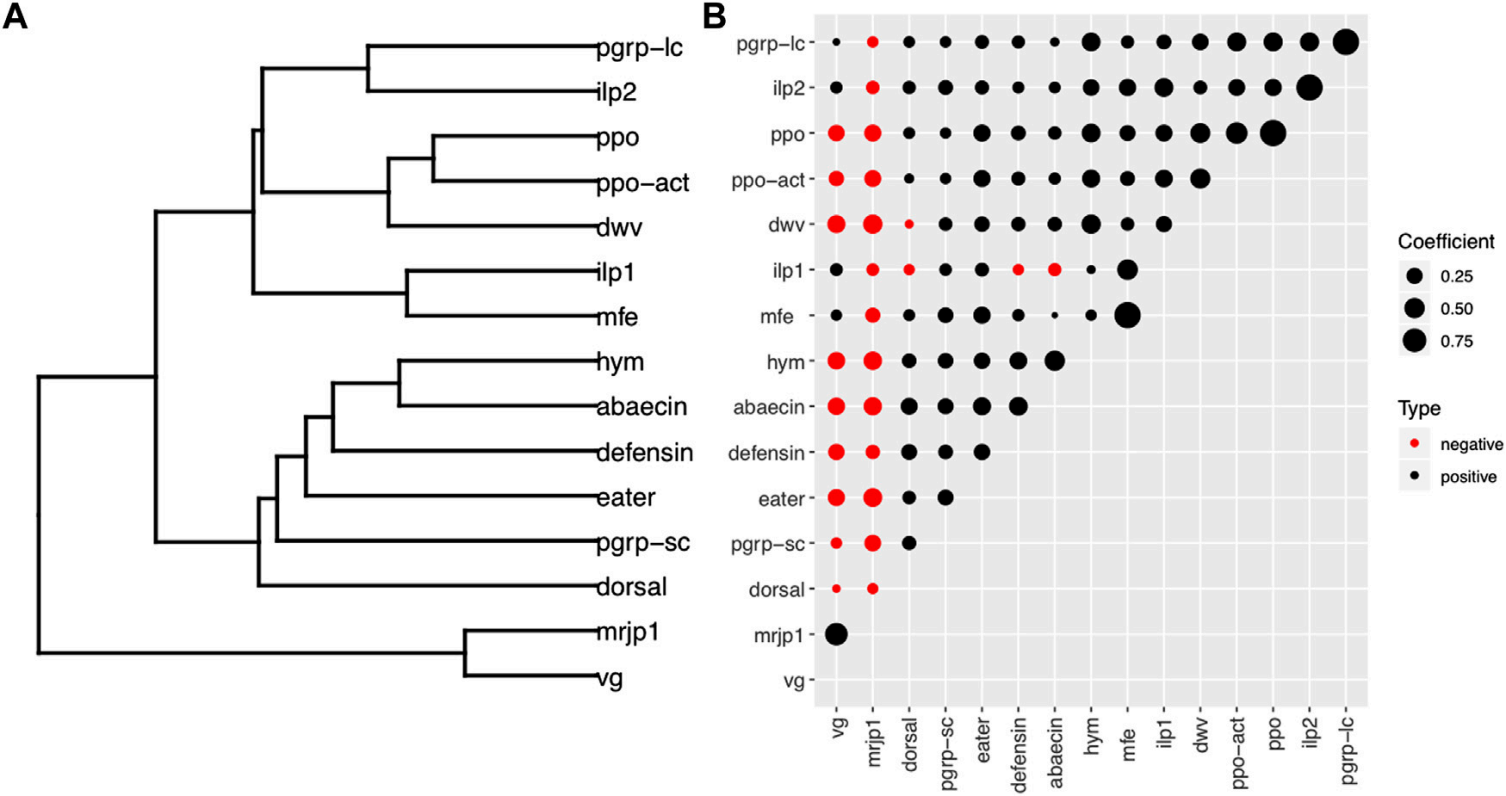
Correlations among physiological genes associated with behavior, immune genes and viruses. Correlations between expression of different genes shown by **(A)** dendrogram and **(B)** correlation matrix. The dendrogram in **(A)** was constructed by hierarchical clustering of a dissimilarity matrix derived from pairwise correlation coefficients **(B)**. The circle size is proportional to the correlation coefficient. Black circles indicate positive correlations; red circles indicate negative correlations. Hierarchical clustering based on Spearman correlations between gene targets defined two major groups. The first cluster consisted of *vg* and *mrjp1*, whose expression is characteristically elevated in nurse bees. The physiological genes associated with foraging, immune genes and DWV formed the second cluster.

**FIGURE 7:**
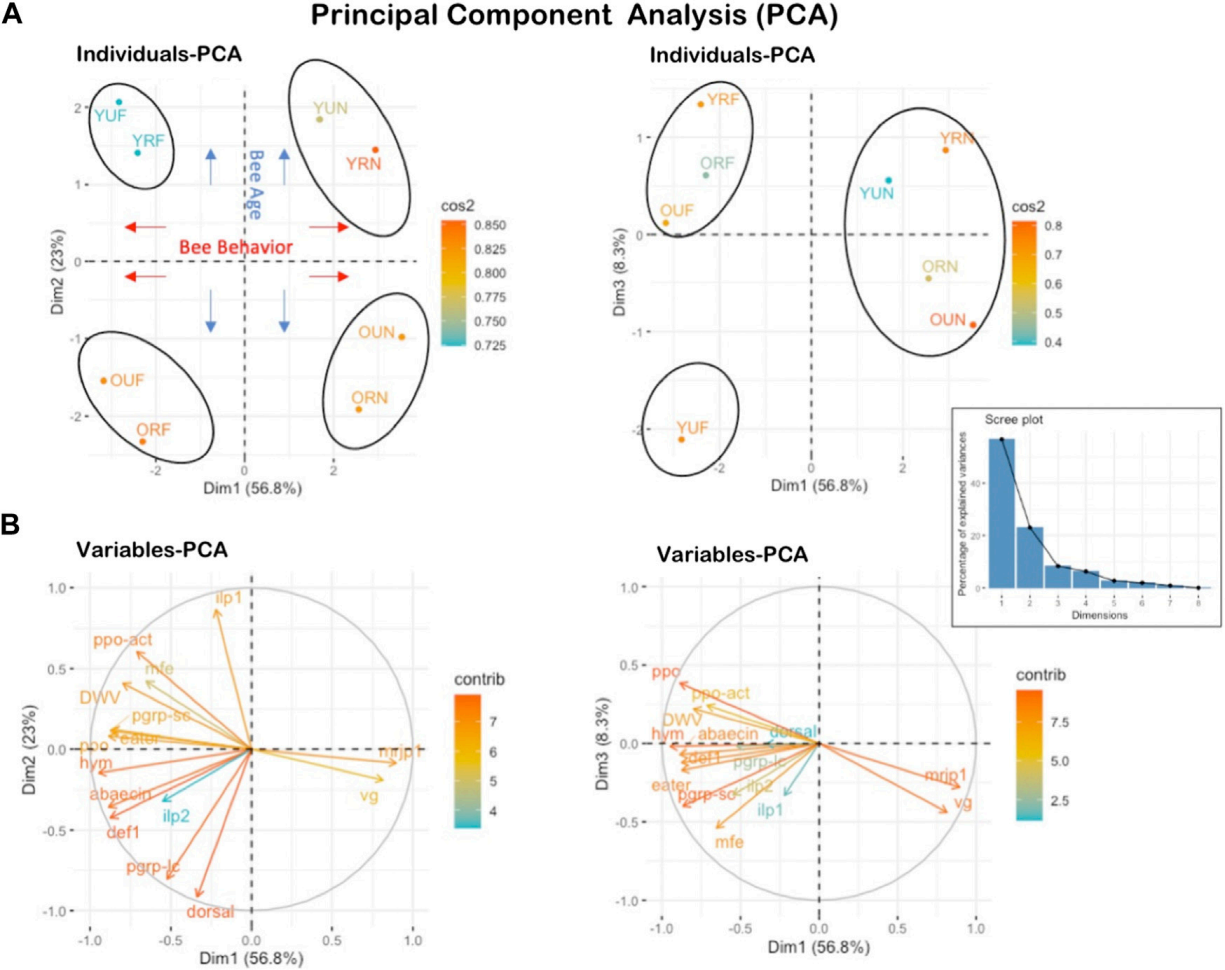
Principal Component Analysis PCA conducted on all studied genes and treatment categories. **(A)** shows the projection of the eight individual treatments (YUF, YRF, OUF, ORF, YUN, YRN, OUN, ORN) on PC axes 1 and 2 as well as 1 and 3.56.8% of the variables are expressed on axis 1% and 23% on axis 2. **(B)** It shows the projection of variables (gene expression) on the three first axes of the PCA. Both *mrjp1* and *vg* genes exhibit opposite expression compared to all other studied genes.

Analyses of the expression of key genes involved in the regulation of honey bee physiology revealed strong positive correlations between the groups of genes encoding storage proteins (*vg-mrjp1,* r *=* 0.664*, p*<0.001), as well as among the group of genes involved in hormonal regulation (*mfe-ilp1 r =* 0.517, *p* < 0.001; *mfe*-*ilp2* r = 0.308, *p* < 0.001) (Supplementary Table 4, Figure 7). Next, we analyzed the relationships between the genes involved in physiological regulation with immune genes. We found a clear difference in the interactions of immune genes and the group of genes regulating physiology. Thus, most immune genes were negatively correlated with *mrjp1* and *vg*, and positively correlated with members of the IIS-JH pathway involved with hormonal regulation (*mfe*, *ilp1*, *ilp2*). Finally, we investigated the relationships between DWV loads with the expression of physiological genes associated with behavior and immunity. Our results showed a remarkable difference in the relationships of DWV with genes involved with physiological regulation, with a highly significant negative correlation with nursing genes and a positive correlation with foraging genes: *mrjp1* (r = −0.424, *p* < 0.001), vg (r = −0.349, *p* < 0.001); *ilp1* (r = 0.249, *p* < 0.001), *ilp2* (r = 0.147, *p* < 0.01). DWV showed a positive correlation with all immune genes but dorsal. The immune genes with the strongest positive correlation with DWV were those involved with the melanization pathway: *ppo-act* (r = 0.477, *p* < 0.001), *ppo* (r = 0.469, *p* < 0.001) (Figure 6). Additional correlation analyses of specific age, nutritional and behavioral groups confirmed the general trends observed in the overall analysis but also revealed significant differences among the experimental groups. Among the most remarkable of them is an important reduction in the number of correlated targets in foragers compared with nurses in colonies not experiencing pollen deprivation (Supplementary Figures 1, 2).

## 6.5 Principal component analysis (PCA)

Rank-based multivariate ordination of gene expression and virus levels revealed the clustering of samples by behavior and age (Figure 7A). Four main clusters were revealed in this analysis, with an overall expression of variables of 56.8% on the first-dimensional axis (Dim1), 23% on Dim2, and 8.3% on Dim3 (Figure 7). The four discriminated clusters in this analysis were: 1- young foragers (YUF, YRF), 2- young nurses (YUN, YRN), 3- old foragers (OUF, ORF), 4- old nurses (OUN, ORN). The physiological behavior was discriminated on Dim1 providing a substantial divergence (56.8%) between nurses and foragers regardless of the pollen restriction status. The second factor of importance is the bee age, which explained 23% of the variance on Dim2. The number of predicted clusters according to the Scree plot (elbow of the graphic line) can range between 3-4 clusters. The third-dimensional axis (Dim3) absorbed 8.3% of the variance and reaffirmed to a great extent the finding of Dim1, which segregated the treatments based on their physiological behavior (nurse vs. foragers) (Figure 7). The PCA conducted on the variables individually (genes) indicates uniqueness and the stark contrast between the overall expression of both *vg* and *mrjp1* (arrows on opposite directions) and the rest of the studied genes (Figure 7B).

## 6.6 Non-metric multidimensional scaling (NMDS)

**FIGURE 8:**
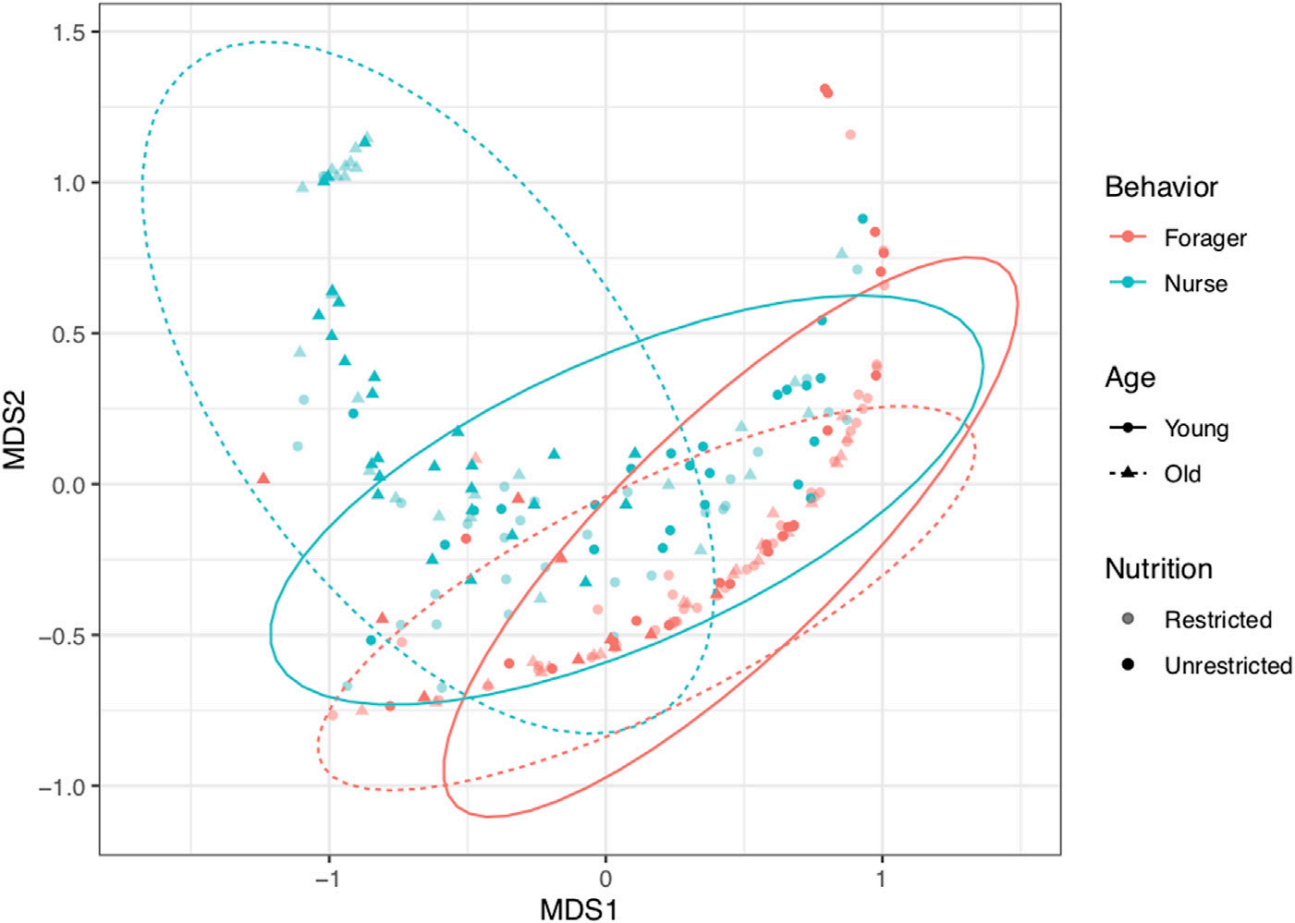
Multivariate ordination of samples based on non-metric multidimensional scaling (NMDS) of gene expression and virus levels. Behavior had the strongest effect on expression patterns (F_1, 197_ = 55.13, *p* < 0.001, *R* ^2^ = 0.19). Age (F_1, 197_ = 22.29, *p* < 0.001, *R* ^2^ = 0.076) and the interactions between age and behavior (F_1, 197_ = 8.75, *p* < 0.001, *R* ^2^ = 0.030) and age and nutrition (F_1, 197_ = 9.02, *p* < 0.001, *R* ^2^ = 0.031) were also significant. Color indicates behavior (red for foragers, blue for nurses). Shape and line type indicate age (circles and solid lines for first collection (2-week-old); triangles and dashed lines for second collection (3-week old). Shading indicates colony nutrition treatment (opaque for pollen-restricted (“R”), solid for unrestricted (“U”). Ellipses show 95% confidence limits for combinations of Behavior and Age.

Rank-based multivariate ordination of gene expression and virus levels revealed the clustering of samples by behavior and age (Figure 8). Young (2-week-old) and old (3-week old) foragers were similar to each other, and more closely resembled young than old nurses. Patterns in the ordination were supported by the results of the permutational MANOVA. Behavior had the strongest effect on expression patterns (F_1, 197_ = 55.13, *p* < 0.001, *R* ^2^ = 0.19), explaining more than twice as much variation as any other treatment. Age (F_1, 197_ = 22.29, *p* < 0.001, *R* ^2^ = 0.076) was the only other term that explained more than 5% of variation among samples. The interactions between age and behavior (F_1, 197_ = 8.75, *p* < 0.001, *R* ^2^ = 0.030) and age and nutrition (F_1, 197_ = 9.02, *p* < 0.001, *R* ^2^ = 0.031) were also statistically significant, but of lower magnitude. In contrast, nutrition alone did not explain significant variation in the response variables (*p* ≈ 1.0). Old nurses, represent the group with the most divergent expression profile, which is mainly derived from the upregulation of *vg*, *dorsal* and humoral immune genes, and low levels of viruses (Figures 2, 3; Supplementary Table 3).

## 7 Discussion

### 7.1 Genes involved in physiological regulation

Our results on *vg* expression provide important information on the effects of behavior, nutrition and age at the colony level. Consistent with previous reports (Piulachs et al., 2003; Corona et al., 2007), our results showed that *vg* expression is higher in nurses compared with foragers. Additionally, we demonstrated that this behavioral effect is independent of nutritional conditions and age. Nutritional comparisons between pollen-restricted and pollen-unrestricted colonies showed that increased pollen availability is associated with higher levels of *vg*. Similar results, showing higher *vg* levels associated with pollen feeding, have been obtained in cage studies (Cremonez et al., 1998; Ament et al., 2008; Di Pasquale et al., 2013; Di Pasquale et al., 2016) as well as in a field colony-level study (Smart et al., 2016b). However, our study is the first to investigate the effect of pollen feeding in nurses and foragers at different ages. The finding that a higher nutrition status increases *vg* expression independently of the age in nurses, but not in foragers, has practical implications for sample collections in studies where *vg* is used as a biomarker of nutrition and health. In addition, our expression analysis revealed a trend toward higher expression levels in older nurses irrespectively of nutritional conditions. The biological meaning of this phenomenon is unknown; however, it is interesting to note that higher levels of *vg* are also found in long-lived winter bees (Fluri et al., 1977). Also, it is noteworthy that old nurses are the group with the most divergent expression profile, which is primarily the consequence of the upregulation of *vg*, *dorsal* and humoral immune genes but low levels of viruses. Thus, this expression pattern could be associated with the longer lifespan observed in this age and behavioral group.

*Mrjp1* expression patterns revealed several insights. First, we found highly significant differences between nurses and foragers independently of nutritional condition and age. This expression pattern unveils *mrjp1* as a reliable biomarker of behavior for whole body analyses. Second, foragers showed intriguing changes of *mrjp1* expression associated with nutrition and age. Younger foragers from colonies with high pollen storages (U) had higher *mrjp1* levels compared with foragers from colonies with low pollen storage (R). Older foragers experienced an increase in both nutritional groups compared with younger foragers. What can be the biological meaning of these results? *mrjp1* is highly expressed in the fat bodies and other tissues and organs besides the hypopharyngeal glands (Drapeau et al., 2006; Corby-Harris et al., 2014; Niu et al., 2014), suggesting that in addition to their traditional role as a secreted protein, *mrjp1* is used endogenously for other nutritionally related functions (Drapeau et al., 2006; Ji et al., 2014; Niu et al., 2014). These results suggest a critical nutritional function of MRJP1 during the regulation of worker behavioral transition. Finally, we found that overall *mrjp1* expression is over 2.5 orders of magnitude higher than *vg.* This result is consistent with the results obtained by RNA sequencing, where it was determined that the most abundant transcripts in the worker honey bee body were *apisimin* and *mrjp1* (Boutin et al., 2015), which encode for proteins that are part of an oligomer in native royal jelly (Tian et al., 2018).

This study provides new information into the dynamics of *mfe* expression in whole-body analysis. First, we found higher levels of *mfe* in foragers compared with nurses in younger workers of colonies not experiencing nutritional stress. However, it is important to note that the magnitude of this difference is lower compared with the results obtained by direct comparison of isolated *corpora allata* from nurses and foragers (Bomtorin et al., 2014). This result suggests that *mfe* expression in other tissues reduces the difference in overall body expression. Consistent with this possibility, *mfe* expression has been detected in the ovaries of nurses honey bee (Niu et al., 2014), and JH synthesis has been reported in the ovaries of beetles (Tian et al., 2010) and mosquitoes (Borovsky et al., 1994).

Our analysis of *ilp1* showed that this gene presented behavioral differences in younger workers from colonies with unrestricted access to pollen, in which foragers had significantly higher levels compared with nurses. Thus, *ilp1* expression patterns in the whole-body followed a similar pattern to that reported in isolated brains, but this similarly is restricted to young individuals of colonies not experiencing nutritional stress. The magnitude of the difference between nurses and foragers for the whole body is lower than that observed in brains (Corona et al., 2007; Ament et al., 2008). This result suggests that *ilp1* expression in young nurses is upregulated in other tissues when the colony is not nutritionally stressed. This explanation is consistent with the report of upregulation of this gene following amino-acid supplementation in fat bodies of 7-day-old bees (Nilsen et al., 2011) and with the observation of higher levels of *ilp1* in the abdomens of young nurses compared with young foragers in non-nutritionally stressed colonies (Lourenço et al., 2019). The expression profile of *ilp2* showed important similarities and differences compared with *ilp1.* As with *ilp1*, significant differences between nutritional groups were observed only in older workers. However, in contrast with *ilp1*, *ilp2* showed higher levels in young foragers in both nutritional conditions. These results, together with previous reports showing differential regulation in tissues such as the brain (Corona et al., 2007; Ament et al., 2008) and fat bodies (Alaux et al., 2011; Nilsen et al., 2011), reveal a high level of complexity in the regulation of these genes. Overall, our whole-body analysis support previous studies showing that insulin like peptides are upregulated in foragers. However, our results revealed that this effect is restricted to young foragers, and in the case of *ilp1,* is depending on pollen consumption.

### 7.2 Immune genes

In most of the comparisons showing expression changes associated with pollen availability, immune gene expression was upregulated by pollen ingestion compared with pollen restriction. Most of the comparisons not showing upregulation by pollen ingestion were observed in a single non-effector regulatory gene (*dorsal*). The results obtained are consistent with the notion that the availability of nutritional resources is required to keep an active immune system (Moret and Schmid-Hempel, 2000). From another angle, our analysis also revealed that *dorsal* was the immune gene most upregulated by nutritional stress. This finding is significant as this is a regulatory gene that activates the expression of effector immune genes belonging to the Toll pathway (Evans et al., 2006; Di Prisco et al., 2013).

Our results suggest that while the activation of immune response after the nurse-to-forager behavioral transition involves both the humoral and cellular immune response, age-dependent changes in their expression are partly behavioral and nutritional dependent, with old foragers being unable to maintain high expression of genes involved in the cellular immune response when experiencing nutritional stress. Bull et al. (2012) reported increased expression of genes involved in the humoral immune response in old foragers compared with 1-day-old bees. These results are consistent with our findings of higher expression of immune genes in foragers and the upregulation of regulatory and effector genes involved in the humoral immune response in older bees. Nevertheless, our results do not support the view of a general upregulation of immune gene expression in older bees, especially those related to the cellular immune response.

### 7.3 Virus levels

A notable result in our study is that the behavioral state had an important influence on viral titers. This behavioral effect was more substantial in the case of DWV, followed by SBV, while BQCV was the virus less affected by behavior. DWV and SBV have higher viral titers in foragers in most of the nutritional and age groups. Our results show that the only nutritional and age groups not showing behavioral differences in any of these viruses are the younger workers from non-pollen restricted colonies. This observation suggests that both nutritional stress and older age contribute to increased viral titers in foragers. The finding of higher DWV levels associated with foraging has important implications for understanding the relationships among nutritional stress, behavioral development and colony losses caused by viral infections. Nutritional stress caused by pollen dearth is a common situation experienced by colonies within intensively cultivated agricultural areas (Di Pasquale et al., 2013; Smart et al., 2016b; Di Pasquale et al., 2016). Pollen deprivation induces precocious foraging, which in turn is associated with accelerated aging and reduced lifespan (Guzmán–Novoa et al., 1994; Janmaat and Winston, 2000; Rueppell et al., 2007; Perry et al., 2015). We suggest the possibility that increased DWV titers associated with foraging could further reduce forager survival and exacerbate the changes in colony demography leading to colony collapse (Perry et al., 2015).

The effect of nutrition on DWV levels has been explored using caged bees in laboratory conditions (DeGrandi-Hoffman et al., 2010; Alaux et al., 2011; Tritschler et al., 2017). Our results reveal pollen availability was positively correlated with higher DWV titers at the colony level, in contrast with an initial report showing that pollen ingestion decreases viral replication (DeGrandi-Hoffman et al., 2010). However, new lines of evidence are consistent with our results. First, colonies supplemented with polyfloral pollen had higher DWV titers but lower *Nosema ceranae* levels compared with colonies that forage on monofloral Eucalyptus pollen (Branchiccela et al., 2019). This result is consistent with experiments showing that DWV-B replication is inhibited when there is co-infection with *Nosema ceranae* (Tritschler et al., 2017), which altogether suggest that competition for nutritional resources reduces viral replication. Second, Alaux et al. (2011) found higher DWV titers associated with pollen feeding in non-varroa parasitized bees. Moreover, these authors also found that pollen feeding further increases viral replication in varroa-parasitized bees already having high DWV levels. These results suggest that nutritional resources can increase DWV replication, and that this effect is even stronger in colonies with medium-high varroa infestation levels and concomitant increased viral loads. Thus, pollen ingestion may contribute to induce over infection when DWV titer reached certain threshold level.

### 7.4 Correlation analysis

Our analysis of significant correlations in expression among genes and viruses revealed clear trends in their interactions. Genes related with physiological activity showed two groups of co-regulated genes associated to nursing (*vg-mrjp1*) and foraging (*mfe-ilp1-ilp2*). Similarly, immune genes showed a general correlation, with particularly strong positive associations among genes belonging to specific immune pathways, such as the genes encoding AMP and genes involved in the melanization pathway. These results confirm the coordinated response of different immune pathways, which are co-regulated similarly, primarily by behavioral-associated physiological changes (Yang and Cox-Foster, 2005). On the other hand, the groups of genes involved with physiological activity showed a clear difference in their interactions with immune genes. Thus, while the genes associated with nursing showed a strong negative correlation with immune genes, the genes associated with foraging displayed the opposite pattern. These results support an association between the genes involved with physiological regulation with the immune system. Our results are consistent with previous reports showing a negative association between certain immune genes with *vg* in studies related to colony survival (Smart et al., 2016b) or immunosenescence (Lourenço et al., 2019). However, to the best of our knowledge, this is the first time where genes associated with nursing and foraging have been found to be differentially associated with the expression of immune genes. The genes involved with physiological regulation also showed differences in their interaction with viruses. In particular, nursing associated genes (*vg*-*mrjp1*) showed a strong negative correlation with DWV, while foraging associated genes (*mfe-ilp1-ilp2*) showed a predominant positive correlation. Finally, our analysis of the interactions among immune genes and honey bee viruses revealed a predominant positive association between the expression of immune genes and DWV levels. In particular, we found strong positive correlations between DWV levels and the expression of the genes involved in the cellular immune response (*ppo*, *ppo-act*). Indeed, several lines of evidence in honey bees and other insects suggest that the melanization pathway is involved in antiviral resistance (Shelby and Popham, 2006; Beck and Strand, 2007; Ryabov et al., 2016). The finding that DWV levels were positively correlated with Varroa mite-mediated suppression of cellular immunity (Yang and Cox-Foster, 2005) provides further support for the role of cellular immunity in resistance to viral infection.

## 8 Concluding remarks

In this study, we used pollen-restricted colonies as a model to study the nutritional conditions occurring in colonies surrounded by intensively cultivated agricultural areas, which typically experience nutritional stress caused by pollen dearth. The results of this study support previous reports and provide novel findings on the effect of nutritional stress on genes involved in physiological activity, immunity, and viral titers. A notable contribution of the present study is the simultaneous quantification of these groups of targets at colony-level controlling the effects of nutrition, behavior, and age in colonies of similar demography, size, and genetic background. Overall, this study provides new information about how nutrition, behavior, and age determine the expression of key genes involved in honey bee physiology and immunity.

## Data Availability

The original contributions presented in the study are included in the article/Supplementary Materials, further inquiries can be directed to the corresponding author.

## Supplementary material

SUPPLEMENTARY FIGURE 1 Correlation matrices for young worker nutritional and behavioral groups. Correlation analyses were conducted at 95% coefficient interval for each of the treatment separately. Non-significant correlations (p > 0.05) are not shown in any matrix graph.

SUPPLEMENTARY FIGURE 2 Correlation matrices for old worker nutritional and behavioral groups. Correlation analyses were conducted at 95% coefficient interval for each of the treatment separately. Non-significant correlations (p > 0.05) are not shown in any matrix graph.

SUPPLEMENTARY TABLE 1 Primer pairs used for quantitative PCR of honey bee genes and viruses. Forward primer (F), reverse primer (R). All primer pairs had an efficiency of approximately 100% in our experimental system, except for *eater* (89%) and *pgrp-sc* (non-determined) (68). Primer efficiencies were tested using a standard curve (SC) with five 1:2 serial dilutions of a concentrated cDNA pool prepared with RNA from adult bees of different ages. This concentrated cDNA (125 ng/ul) was diluted 4X to obtain the highest concentration point on the SC (31.25 ng/ul). The second point on the SC (15 .6 ng/ul) was used as a reference sample across all the multiple plates and targets analyzed using the *Gene Study* software (Life technologies).

SUPPLEMENTARY TABLE 2 Effects of behavior, nutrition and age on gene expression and viral titers. Differences between groups of variables fulfilling the assumptions of parametric statistics were analyzed with ANOVA. Non-parametric variables were analyzed by Mann Whitney test. Significant *p*- values are indicated in red case.

SUPPLEMENTARY TABLE 3 Average relative values (ARVs) of amplified targets (genes and viruses). Values ARVs were calculated relative to the housekeeping gene *Rps5*, taking in consideration all the behavioral/nutritional/age groups (Total) or each of the different combination of variables. Abbreviations: Nurses (N) Foragers (F); young (Y) vs. old (O), pollen-unrestricted (U) vs. pollen restricted (R) (e.g., pollen unrestricted nurses = Y-U-N). Expression ratios were obtained dividing the ARVs from each pair of variables and used for calculation of differentially amplified targets. For each pair comparison, targets were sorted out with the higher ARVs values and ratios on the top. Single variable: A2 (behavior). B1 (age), B2 (nutrition). Double variable: C (age-behavior), D (behavior-nutrition), E (age-nutrition) F (age-behavior). Triple variable: Nutritional comparisons (G, H), Age comparisons (I, J), behavioral comparisons (K, L).

SUPPLEMENTARY TABLE 4 Correlations among genes and viruses. Correlations in the expression level of the different genes and viruses were analyzed by the Spearman Rank Correlation Test. Positive and negative correlations are indicated in green and red case, respectively. Significant *p*- values are indicated in bold case.
