# Supplementary figures and images for: Decoupling the effects of nutrition, age, and behavioral caste on honey bee physiology, immunity, and colony health
Source: Front Physiol. 2023 Mar 13;14:1149840. doi: 10.3389/fphys.2023.1149840 (PMC10040860; doi:10.3389/fphys.2023.1149840)

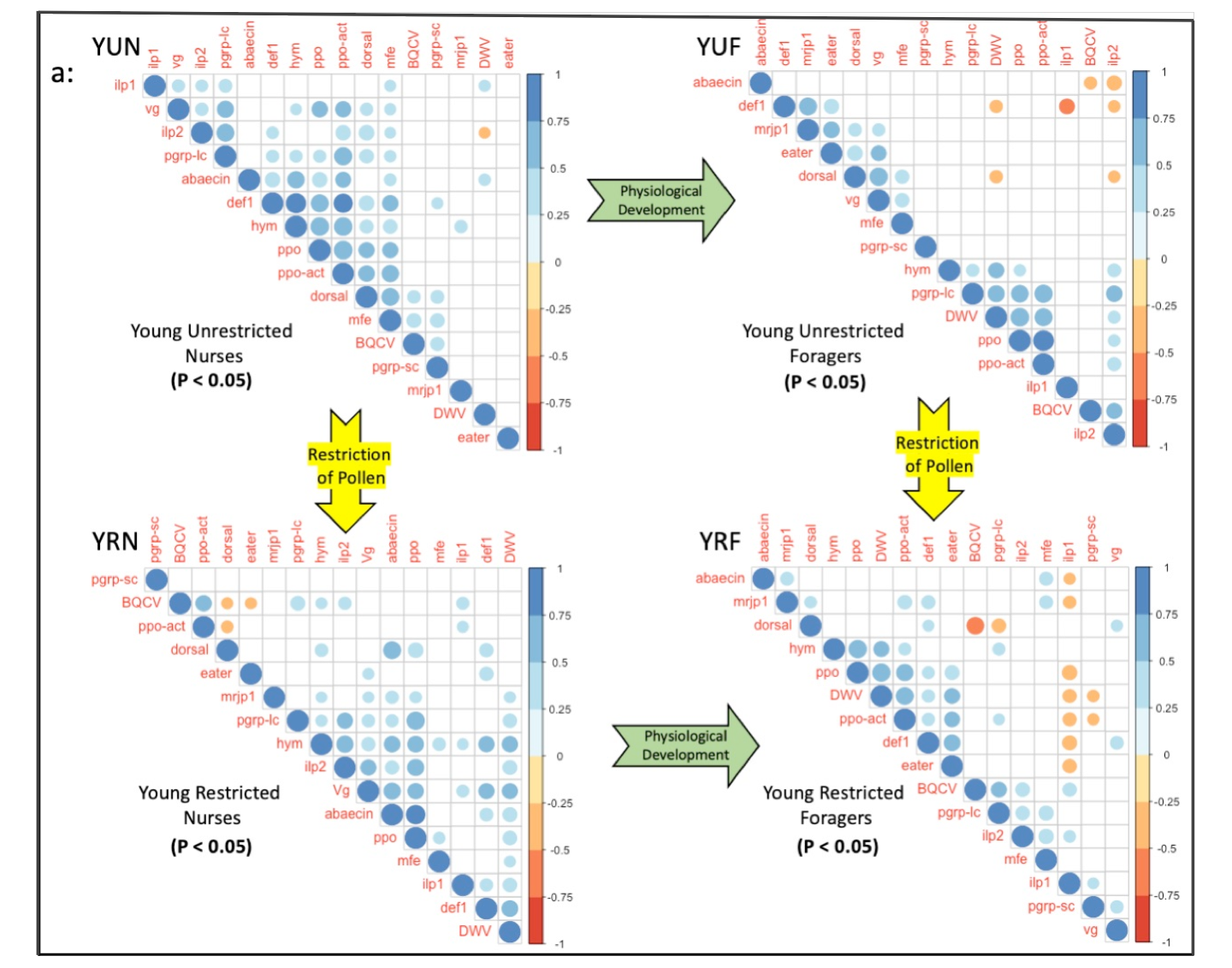

Supplement: Supplementary file 2 [file Image1.TIFF]

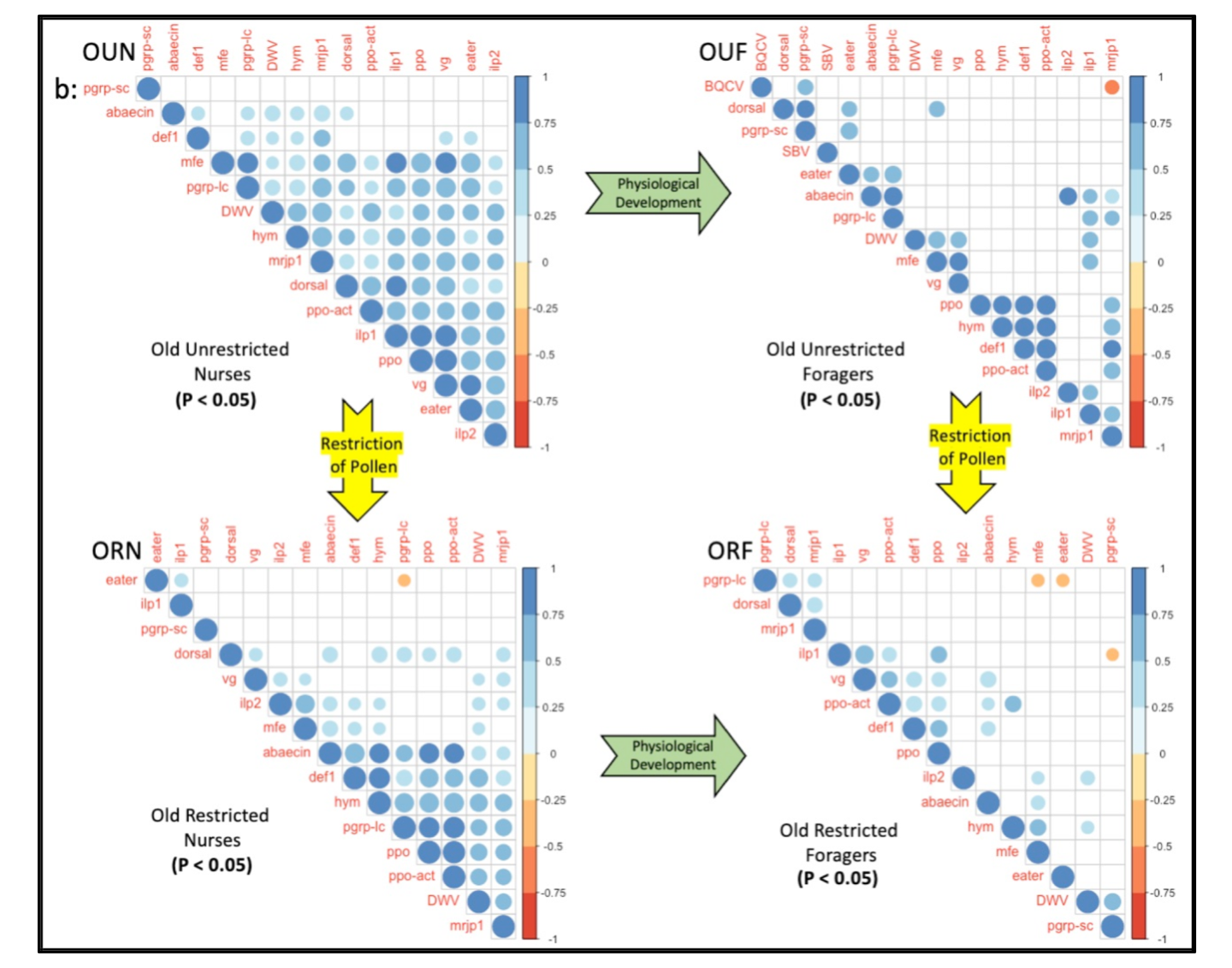

Supplement: Supplementary file 6 [file Image2.TIFF]
